# The Therapeutic Role of Interleukin-1 Inhibition in Idiopathic Recurrent Pericarditis: Current Evidence and Future Challenges

**DOI:** 10.3389/fmed.2017.00078

**Published:** 2017-06-12

**Authors:** George Lazaros, Katerina Antonatou, Dimitrios Vassilopoulos

**Affiliations:** ^1^1st Department of Cardiology, School of Medicine, National and Kapodistrian University of Athens, Hippokration General Hospital, Athens, Greece; ^2^Joint Rheumatology Program, Clinical Immunology-Rheumatology Unit, 2nd Department of Medicine and Laboratory, School of Medicine, National and Kapodistrian University of Athens, Hippokration General Hospital, Athens, Greece

**Keywords:** pericarditis, therapeutics, interleukin-1beta, hereditary autoinflammatory diseases, interleukin-1 receptor antagonist protein

## Abstract

Recurrent pericarditis is a common complication of acute pericarditis (15–30%) for which, in most cases, no underlying etiology is found [idiopathic recurrent pericarditis (IRP)]. IRP is currently viewed as an autoinflammatory disease with characteristic recurrent episodes of sterile inflammation. According to the most recent Guidelines, the initial treatment regimen consists of a combination of aspirin or non-steroidal anti-inflammatory drugs with colchicine followed by the addition of corticosteroids in resistant or intolerant cases. Despite this treatment approach, a number of patients either do not respond or cannot tolerate the above therapies. For this refractory group, small case series and a recent randomized controlled trial have shown that interleukin-1 inhibition with anakinra is a rapidly acting, highly efficient, steroid-sparing, and safe therapeutic intervention. In this perspective, we discuss the available clinical evidence and our own clinical experience as well as the future prospects of this novel therapeutic approach for patients with IRP.

## Introduction

Recurrent pericarditis is a relatively common complication (15–30%) of acute pericarditis (1, 2). Usually occurs after a symptom-free interval of more than 4 weeks after the initial episode of acute pericarditis and may last for years (1, 2). Although in approximately 20% of patients, an initial viral etiology can be documented, in the majority of patients with recurrent pericarditis, no specific cause is identified and the disease is referred as “idiopathic recurrent pericarditis” (IRP) (1, 2).

Established therapeutic regimens like non-steroidal anti-inflammatory drugs (NSAIDs), colchicine, and corticosteroids induce remission and cure in the majority of cases (1, 3). Nevertheless, a subset of patients either does not respond, relapse, or cannot tolerate the above therapeutic interventions (1, 3). In such patients, the disease usually follows a chronic, relapsing course posing a therapeutic challenge for treating physicians.

In this perspective, we review recent data regarding the pathogenesis of IRP and the therapeutic role of interleukin-1 (IL-1) inhibition in this disease. We present critically the available literature data on anakinra, since it is the only IL-1 inhibitor that there is sufficient clinical experience for its efficacy in this disorder.

## Is IRP an Autoimmune or Autoinflammatory Disease?

In the absence of a suitable animal model, there are limited data regarding the pathogenesis of IRP (4). Although in a proportion of patients, non-specific (such as antinuclear antibodies) (5) or organ-specific (anti-heart) (5) autoantibodies have been detected, a pathogenic role for these antibodies has not been proven. Furthermore, autoreactive T-cells directed against pericardial autoantigens have not been identified so far, pointing away from an autoimmune etiology in the majority of patients with this disease.

On the other hand, there are strong indications that IRP could actually be an autoinflammatory disease (6). Autoinflammatory diseases, as proposed by experts (7, 8), are characterized by recurrent episodes of sterile inflammation in different target organs (most commonly skin, serosal surfaces, joints) due to an abnormal activation of the innate immune system provoked by different exogenous (bacteria, viruses, cold exposure) or endogenous (uric acid or calcium pyrophosphate crystals) stimuli.

Certain mutations in genes coding for key signaling proteins of innate immune pathways have been identified such as mutations of the MEFV gene coding for the pyrin protein in familial Mediterranean fever (FMF), the TNFRSF1A gene coding for the tumor necrosis factor receptor 1 protein in tumor necrosis factor receptor receptor-associated periodic syndrome (TRAPS), the MVK gene coding for mevalonate kinase in Hyper IgD syndrome (HIDS), and the NLRP3 gene coding for the NLRP3 protein in the cryopyrin-associated periodic syndromes (CAPS). These diseases are referred as monogenic autoinflammatory diseases (7), while for others (referenced as polygenic), a number of different genes have been implicated (including adult onset Still’s disease-AOSD, Crohn’s disease, sarcoidosis, etc.) (8). Nevertheless, in a number of autoinflammatory diseases, no specific gene perturbations have been identified so far.

A distinctive feature of certain autoinflammatory diseases is the increased production of IL-1β (usually due to dysregulated activation of the NLRP3 inflammasome), which is considered a key mediator of the characteristic clinical manifestations of these disorders (9). Certain autoinflammatory diseases respond rapidly to IL-1 inhibition indicative of the central pathogenic role of this cytokine in these disorders (10).

Idiopathic recurrent pericarditis is currently considered an autoinflammatory disease based on its distinctive features such as its relapsing course with episodes of fever and high inflammatory markers [C-reactive protein (CRP), erythrocyte sedimentation rate (ESR)] in the absence of specific autoantibodies or autoreactive T cells, its serosal involvement (pericardial and occasionally pleural), and its response to colchicine and, more importantly, to IL-1 inhibition (6, 10, 11). The role of IL-1 in the pathogenesis of IRP has been based so far only on clinical data (see text below).

In a small proportion of patients (10%), familial clustering has been observed (12), while a weak association with certain HLA genes such as the DQB1*0202 has been shown (13, 14). Recurrent pericarditis can occur in monogenic autoinflammatory diseases like FMF (18%) (15) or TRAPS (7%) (16, 17), and these diseases account for a small proportion of cases with IRP (18). In a recent study (18), 6% of patients with IRP had TRAPS mutations (most had the low penetrance mutation R92Q). The majority of these patients had a positive family history of pericarditis and/or recurrent fever syndromes and all were resistant to colchicine (18).

These data emphasize the need for genetic screening in the appropriate clinical setting (young patients or adults with a positive family history for pericarditis or autoinflammatory diseases).

## Natural Course

The natural course of IRP is characterized by recurrent episodes of pericardial inflammation after the initial acute pericarditis attack (1, 2). Factors that predispose to the development of IRP include persistently high CRP levels, early use of corticosteroids, an incomplete initial response to NSAIDs, and non-administration of colchicine (2). Among different potential biomarkers, serum IL-8 presence at baseline was associated with a more frequent transition from acute to recurrent pericarditis (19).

Although IRP has an overall favorable prognosis (20, 21), it may last for years with recurrent episodes of chest pain and fever, impairing patients’ daily quality of life while for patients who require long-term corticosteroids, a number of side effects can occur including cataracts, hyperglycemia, cushinoid appearance, osteoporotic fractures, increased risk of infections, etc.

## Current Therapeutic Algorithm for the Treatment of Recurrent Pericarditis

**Figure 1:**
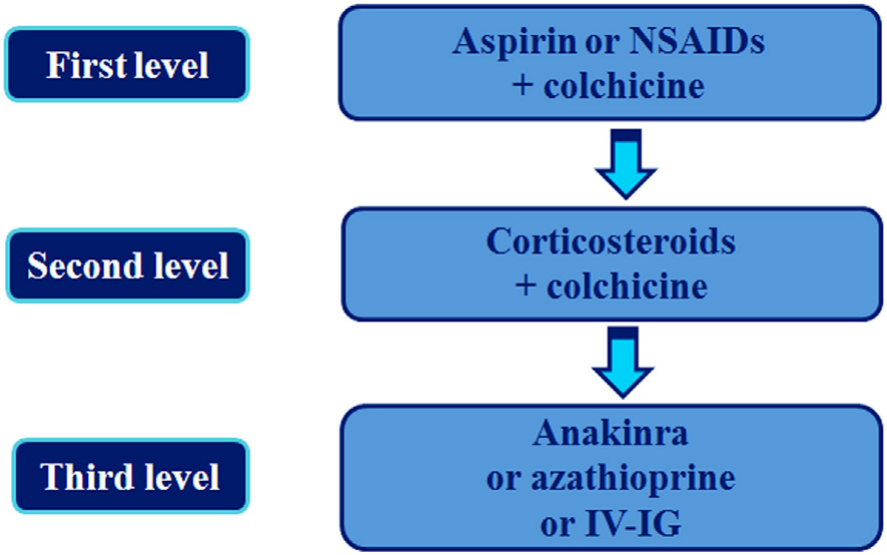
Stepwise pharmacologic approach of the treatment of recurrent pericarditis. This is based on the 2015 European Society of Cardiology Guidelines for the management of recurrent pericarditis [(3), see text for details]. IVIG, intravenous human immunoglobulin; NSAIDs, non-steroidal anti-inflammatory drugs.

The current approach to the treatment of recurrent pericarditis, according to the most recent Guidelines from the European Society of Cardiology (ESC) (3) is shown in Figure 1.

### NSAIDs–Colchicine–Corticosteroids

The initial therapy consists of a combination of aspirin (ASA) or NSAIDs (at therapeutic doses, i.e., ibuprofen 600 mg ×3, indomethacin 25–50 mg ×3) and colchicine (0.5 mg × 2 pos) (3). In patients who do not respond or cannot tolerate ASA/NSAIDs, corticosteroids are administered as a second line treatment (prednisone or equivalent 0.2–0.5 mg/kg/day pos). For patients who display a complete response (absence of symptoms with a normal CRP), NSAIDs and corticosteroids are gradually tapered and discontinued followed by tapering and discontinuation of colchicine (given at least for 6 months).

### Azathioprine (AZA)—Intravenous Immunoglobulins (IVIG)

Patients who fail to obtain remission with the second level approach or do so with unacceptably high doses and those who are intolerant to this regimen fall into the entity of refractory IRP. This entity is among the most challenging in the field of pericardial diseases and encompasses patients who require high doses of steroids (>10 mg/day of prednisone) in order to control disease flares (3, 22, 23). It is estimated that approximately 5% of recurrent pericarditis cases belong to this group (22). The third level approach actually addresses this problematic subgroup of patients with refractory pericarditis. Treatment options in this step include anakinra, AZA, and IVIG.

All above therapeutic options received a IIb class of recommendation with a level of evidence C, which means that such suggestion is based on experts’ opinion. The selection of either agent should rely on the individual patient characteristics, local expertise, and cost.

For instance, AZA, as already mentioned, is not suitable for the acute phase since it is a slow acting agent useful to control disease activity in the long run, allowing steroid tapering or discontinuation (3). The largest published series until today on AZA administration in IRP consists of one retrospective, single-center study of 46 patients with IRP (24). Among them, 85% were considered as responders and were able to discontinue corticosteroids between 4 and 12 months of AZA commencement. Disease remission was achieved in ~59% with AZA but at the end of follow-up period, 24% of patients were still requiring AZA (24). Side effects like transient hepatotoxicity (11%) and leukopenia (6.5%) were not uncommon.

Human IVIG has the potential advantage of an immunomodulatory rather than an immunosuppressive effect (25). However, the available experience is very limited consisting of only 30 cases (summarized in a recent systematic review) (25). Notably, only half of them suffered from the idiopathic form while the rest had either an autoimmune or infectious cause. IVIG was administered at a dose of 400–500 mg/kg/day for five consecutive with the possibility to repeat treatment, usually a month later, in case of a partial clinical response (25). In a 33-month follow-up period, 73% of patients were free of recurrences with only 17% still receiving steroids. Overall, IVIG had a rapid onset of action and was an efficient steroid-sparing agent. Its high cost, the limited clinical experience, and the need for inpatient hospital administration are matters of concern.

### Anakinra

#### Review of Anakinra Clinical Data in IRP Patients

Anakinra is a recombinant IL-1 receptor antagonist that inhibits the action of IL-1, which is already approved for the treatment of rheumatoid arthritis (RA) for more than 15 years and has been successfully used off label for the treatment of various monogenic (FMF, TRAPS, CAPS, HIDS) (26), polygenic (systemic onset juvenile idiopathic arthritis, AOSD, gout, calcium pyrophosphate deposition disease) (26) or of undefined etiology (27) systemic autoinflammatory diseases.

**Figure 2:**
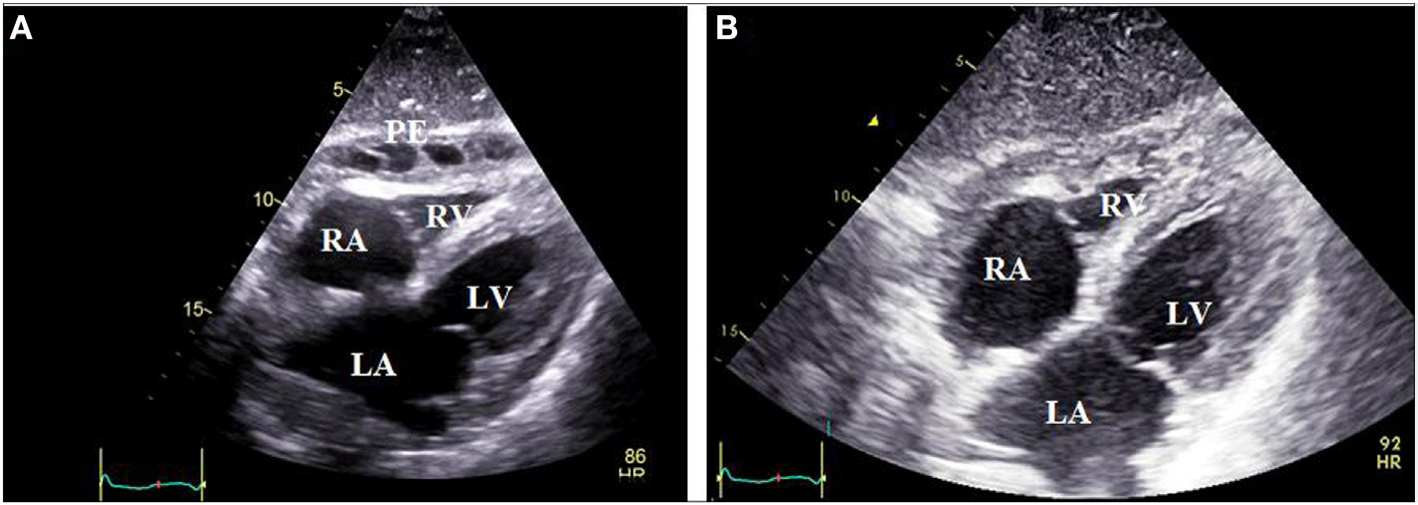
Subxiphoid echocardiographic view of a patient with colchicine-resistant steroid dependent idiopathic recurrent pericarditis. Panel **(A)** depicts pericardial effusion mainly in the anterior pericardial space with thick adhesions between visceral and parietal pericardium, which developed during the steroid tapering process. Panel **(B)** reveals absence of effusion in the same patient 1 month after switching from steroids to anakinra therapy (100 mg subcutaneously daily). PE, pericardial effusion; LA, left atrium; RA, right atrium; LV, left ventricle; RV, right ventricle.

Following the first case series of pediatric (6) and adult (28, 29) patients with IRP who were treated successfully with anakinra, a long-term cohort study including 10 patients was reported in 2014 (30). Anakinra proved highly effective, achieving symptoms relief within 2 days, rapid decrease in acute phase reactants (ESR/CRP), and pericardial inflammation (Figure 2). Most importantly, anakinra administration allowed steroid discontinuation in all patients within approximately 38 days without any serious adverse effects. Anakinra was administered initially for 6 months at a dose of 100 mg subcutaneously (SC) followed by alternate day administration for six additional months. Using this protocol, though a high recurrence rate after drug discontinuation (70%) was observed.

All the available current evidence on anakinra administration in IRP cases was assessed in a systematic review of the published evidence (four case series and five case reports) until October 2014 (11). The total number of patients was 34 with a mean age of 26.8 years. All patients had a long-standing disease of approximately 31 months duration with several recurrences and were either resistant to all available regimens (colchicine was given as baseline treatment in ~80% of cases) or unable to tolerate them. In all, but one, case, the administered daily dose was 100 mg SC for 9 months on average and in approximately two-third of cases, dose tapering was adopted before drug discontinuation. Concerning efficacy, anakinra achieved an immediate disease control leading to symptom remission and CRP normalization within 1 week. This is an advantage in comparison to AZA, which requires several weeks to control disease activity (24). Corticosteroids were rapidly tapered and finally discontinued within approximately 2 months in all patients. Anakinra controlled disease activity in all circumstances during the full-dose regimen. Recurrence was recorded either during dose tapering in 26% of patients or after drug discontinuation. During follow-up (~28 months), almost one out of four patients (23.5%) were disease and treatment free.

Similar to adults, a number of children with IRP have been treated successfully with anakinra (6, 31, 32). The anakinra dose was 100 mg/day for adults (30, 33, 34) and 1–2 mg/kg/day (up to 100 mg/day) for children (3, 31, 33).

Regarding safety, adverse reactions requiring drug discontinuation were recorded in only 3% of cases and consisted of reversible transaminasemia in one patient. The most common side effect was a local injection reaction (~44%), usually during the first month of treatment. In agreement with the long-term safety data from RA patients treated with anakinra (35), no serious side effects were observed in this patient population (11).

Prior to anakinra initiation, screening for hepatitis B virus infection and latent tuberculosis should be performed (36, 37) and patients with positive testing should be managed according to existing guidelines (38). Anakinra should not be initiated in patients with active infection or pre-existing malignancy (36).

Based on these preliminary positive results, anakinra was included as an alternative therapeutic option to AZA and IVIG for patients with resistant to NSAIDs, colchicine, and corticosteroids IRP, in the most recent ESC Guidelines (3).

The efficacy and safety of anakinra was recently evaluated in the first ever randomized, double-blind, placebo-controlled withdrawal trial in refractory cases of IRP: the Anakinra—Treatment of Recurrent Idiopathic Pericarditis trial (AIRTRIP) (33). The trial included 21 consecutive patients with at least three recurrences provided that they had CRP elevation, resistance to colchicine, and need for corticosteroid administration to achieve remission. All enrolled patients received open-label anakinra for 2 months (2 mg/kg/day, to a maximum of 100 mg SC). After that time period, all patients who reached remission with anakinra were randomized to two groups: one group continued anakinra (11 patients) and a second was switched to placebo for the next 6 months. All patients were able to discontinue corticosteroids within 6 weeks while more than half (12/21, 57%) continued colchicine during the double-blind withdrawal period.

During a median follow-up period of 14 months, recurrence of pericarditis was recorded in 90% (9/10) of patients on placebo compared to only 18% of patients (2/11, at 33 and 120 days after randomization) on anakinra (33). The time elapsed between randomization and recurrence onset was 72 days in the placebo group, whereas it was not calculable in the anakinra group (*p* < 0.001). As far as safety is concerned, local injection reactions were again observed during the first month of anakinra administration in all but one patients. Other side effects included one case of herpes zoster and three cases of transient transaminasemia. None of these side effects led to permanent drug discontinuation.

Canakinumab is the only IL-1 inhibitor approved for the treatment of various autoinflammatory diseases including FMF, TRAPS, HIDS, CAPS, AOSD, systemic juvenile idiopathic arthritis, and gouty arthritis (39–41). Canakinumab has the advantage of being administered less frequently (2–4 mg/kg every 4–8 weeks SC) (39). So far, there has been only one case report of unsuccessful use of canakinumab in a child with IRP who finally responded to anakinra (42). Obviously, more studies regarding the use of canakinumab in IRP are needed.

#### Unresolved Issues with Anakinra Administration in IRP Patients

Collectively, the acquired clinical experience with anakinra, so far, indicates that it is a highly effective, rapidly acting, steroid-sparing, and relatively safe agent for the treatment of IRP refractory to NSAIDs, colchicine, and corticosteroids. Even though our experience with anakinra in patients with refractory IRP is continuously increasing, certain issues remain to be answered regarding its more appropriate use in daily clinical practice.

The first important issue is to define the exact anakinra administration protocol. AIRTRIP trial offers a starting point with the 8-month protocol applied (33). However, the duration of this initial treatment period is purely empirical. Patients with long-standing disease and multiple recurrences may require longer periods of anakinra administration before drug tapering and discontinuation is attempted. Although disease remission criteria have not been clearly defined, before any attempt of anakinra dose tapering or discontinuation, the patient should be asymptomatic, without electrocardiographic or echocardiographic evidence of disease and with persistently normal CRP levels. Cardiac MRI, which can detect subclinical pericardial inflammation (edema and late gadolinium enhancement), has been shown recently to be a useful prognostic marker for recurrent disease (43) and a valuable tool in treatment decisions (21). Whether cardiac MRI could assist in designing the appropriate treatment strategy in IRP patients treated with anakinra remains to be proven.

Another important issue to address is the way of anakinra discontinuation in patients with IRP who achieve remission. Although until today, there are no studies directly comparing abrupt drug withdrawal to gradual dose tapering with respect to recurrences, most experts recommend a gradual dose tapering (11, 44). From our own group experience of 11 patients treated with anakinra for approximately 3 years, three (27.5%) were able to discontinue all therapies while almost half (5/11, 45%) could be maintained on a reduced dose (1–4 times a week) (45). Switching from three to two injections a week was a critical point for pericarditis relapse in our cohort (45). No additional safety issues were observed during long-term follow-up. Certainly, more efficient ways of Anakinra tapering are needed in order to achieve treatment discontinuation in all patients.

An additional issue needing clarification is the utility of colchicine coadministration in patients treated with anakinra. Colchicine was given during the open-label phase in all but three patients in the AIRTRIP trial and no difference in the rate of relapses was seen between patients who continued (7/12, 58%) or stopped (4/9, 44%) colchicine during the double-blind withdrawal period (33). Whether colchicine confers additional benefit beyond anakinra is unclear at the moment.

## Future Perspectives

Anakinra is the first biologic agent that has been successfully tried in patients with refractory IRP. Although the available data are derived from a small number of patients, this is the first agent ever tested in a randomized controlled trial (RCT) for this disease. The open label and RCT data have clearly demonstrated that anakinra is a rapidly acting, steroid-sparing, and safe agent for the majority of patients with refractory to NSAIDs, colchicine, and corticosteroids disease. Based on the presented scientific evidence, we believe that it has a strong therapeutic advantage compared to existing alternative therapies such as AZA or IVIG.

Anakinra may also have a role in patients who have failed NSAIDs and colchicine and have contra-indications to corticosteroid use (history of diabetes, osteoporotic fractures, recurrent infections, etc.). This is particularly the case for patients with symptomatic disease and high inflammatory burden where a rapid therapeutic response is needed.

Important issues that remain to be studied further is the appropriate duration of the initial treatment, its tapering protocol, and the requirement for colchicine coadministration. The high cost of the medication should be also taken into account in therapeutic decisions.

Besides its obvious clinical consequences, the use of an IL-1 inhibitor (anakinra) in the treatment of IRP could help us understand better the pathogenesis of an inflammatory disease of unknown etiology.

In conclusion, IL-1 inhibition appears to be one of the most promising strategies for the treatment of IRP that requires further studies in order to establish its exact place in the therapeutic algorithm.

## Author Contributions

All authors participated in the conception, drafting, and final approval of the manuscript and agreed to be accountable for all aspects of the work in ensuring that questions related to the accuracy or integrity of any part of the work are appropriately investigated and resolved.

## Conflict of Interest Statement

The authors declare that the research was conducted in the absence of any commercial or financial relationships that could be construed as a potential conflict of interest.
